# Identification of a new goat torovirus strain: first detection and genomic analysis in China

**DOI:** 10.1186/s13620-025-00305-3

**Published:** 2025-08-27

**Authors:** Kegu Ji’e, Falong Yang, Yang Su, Keha-mo Abi

**Affiliations:** 1 College of Animal & Veterinary Sciences, Southwest Minzu University, Chengdu, 610041 PR China; 2 Key Laboratory of Qinghai - Tibetan Plateau Animal Genetic Resource Reservation and Utilization, Southwest Minzu University, Chengdu, 610041 China

**Keywords:** Goat, Torovirus, Novel, Epidemiology, Metagenomics

## Abstract

Using viral metagenomics, we identified a novel torovirus, GToV/SWUN/SC, in diarrheal goat fecal samples with a genome length of 28,457 nt. This strain shares 96.73–96.79% nucleotide identity with Antelope torovirus (AToV) but only 88.43% with the GToV/SZ strain. Phylogenetic analysis revealed a close evolutionary relationship between GToV/SWUN/SC and AToV. Structural analysis showed three distinct structural variations in the HE protein and multiple amino acid mutations in the S gene, which may influence host adaptation. RT-PCR detected a 35.9% (240/669) positivity rate, indicating widespread circulation of GToV in Sichuan, Chongqing, and Yunnan. This study enhances the understanding of torovirus epidemiology and evolution, providing a theoretical basis for further research on viral diversity.

**Supplementary Information:**

The online version contains supplementary material available at 10.1186/s13620-025-00305-3.

## Introduction

Toroviruses belongs to the genus Torovirus, subfamily Torovirinae, family Tobaniviridae, order Nidovirales (https://ictv.global/taxonomy). It is an enveloped, positive-sense, single-stranded RNA virus with a genome size of 28–29 kb. ICTV classifies toroviruses into four species: Bangali torovirus (Bangali torovirus, BaToV), Torovirus bovis (Bovine torovirus, BToV), Torovirus equi (Equine torovirus, EToV), and Torovirus suis (Porcine torovirus, PToV), while unclassified members include Goat torovirus (GToV), Antelope torovirus (AToV), Guangdong chinese water snake torovirus, Guangdong mandarin rat snake torovirus, Guangdong red-banded snake torovirus, Guangxi torovirus, Henan torovirus, Hubei torovirus, Human torovirus and Torovirus sp. (ToV sp.) (https://www.ncbi.nlm.nih.gov/Taxonomy/Browser/wwwtax.cgi? mode=Undef&id=11155&lvl=3&lin=f&keep=1&srchmode=1&unlock). Among them, Human torovirus was formerly classified as a species within the genus *Torovirus*, according to the 2017 release of the virus taxonomy (MSL #32: https://ictv.global/taxonomy/visual-browser).

Currently, only one complete coding sequence of Goat torovirus (GToV) is available in GenBank — GToV/SZ (accession number: KR527150.1), which was submitted from Hong Kong, China in 2017. AToV was identified in Tibetan antelope in 2021 [4]. The torovirus genome contains six open reading frames (ORFs) encoding key structural and replication proteins, with the spike (S) and hemagglutinin-esterase (HE) proteins playing roles in viral entry and host interactions [5, 7, 13, 21, 30, 32]. Toroviruses mainly infect ungulates, causing diarrhea, especially in horses, cattle, and pigs [4]. Studies have found neutralizing activity against EToV in the sera of animals such as cattle, goats, sheep, pigs, rabbits, and mice, providing serological evidence for the presence of torovirus in other animals [2, 3, 20, 40]. BToV has been linked to diarrhea in cattle of all ages [1, 11, 13], while PToV shows a high detection rate in diarrheic samples [15, 34]. Despite global detection of BToV and PToV [16, 32, 36], research on GToV remains limited.

Although toroviruses have been reported in various animal species such as cattle, pigs, and horses, there are currently no published studies on the detection of toroviruses in goat fecal samples. Therefore, this study aimed to investigate the presence of Goat torovirus (GToV) in diarrheic fecal samples collected from goats in Sichuan, Yunnan, and Chongqing provinces of China using high-throughput sequencing and RT-PCR. The findings will provide fundamental data for the epidemiological and molecular characterization of GToV, contributing to future surveillance and control strategies.

## Materials and methods

### Sample collection and RNA extraction

**Fig. 1:**
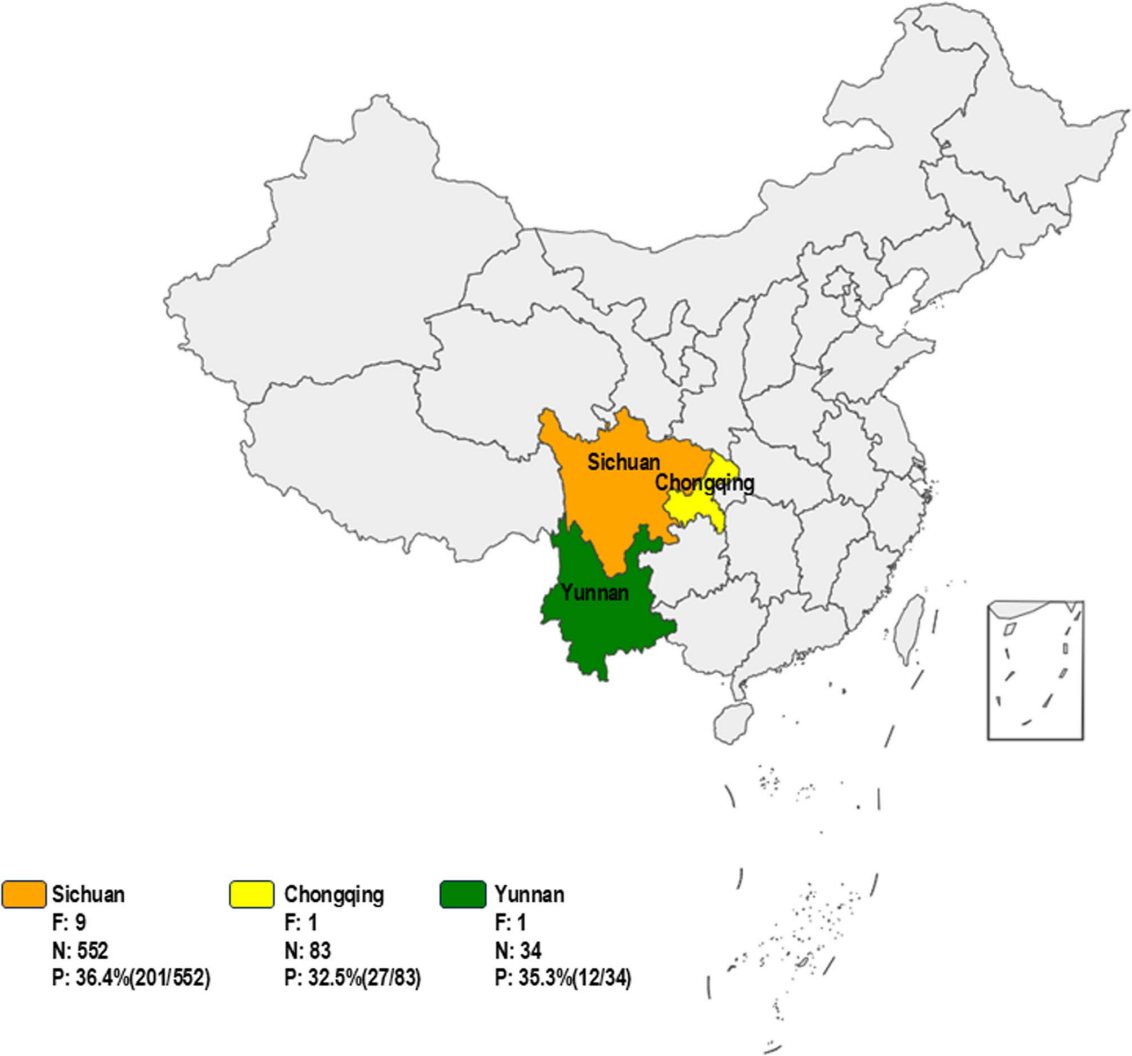
Number and positivity rate of samples from three regions in China, 2020–2025. Note: F represents the number of goat farms, N represents the number of fecal samples, and P represents the positivity rate of GToV

From 2020 to 2025, 669 diarrheal fecal samples were collected from goats in Sichuan, Chongqing, and Yunnan (Fig. 1). Samples were processed, stored at −80 °C, and RNA was extracted using the QIAamp Virus Mini Kit (QIAGEN). cDNA synthesis followed the manufacturer’s instructions using the ExonScript RT SuperMix with dsDNase (Exongen Biotech Co., Ltd.).

### High-throughput sequencing

Three diarrheal fecal samples from different regions were pooled for total nucleic acid extraction, followed by whole-virome library construction and sequencing on the Illumina NovaSeq 6000 platform. After sequencing, raw data were processed with BBDuk (https://sourceforge.net/projects/bbmap/) to perform quality control by removing low-quality reads, reads with high proportions of ambiguous bases (N), and adapter sequences, resulting in clean reads. The quality of clean reads was assessed using FastQC (https://www.bioinformatics.babraham.ac.uk/projects/fastqc/). Clean reads were then mapped to the host genome using Bowtie2 [22] to eliminate host-derived sequences, generating effective reads. Kraken2 [41] was used to align effective reads against the RefSeq database (including genomes of humans, bacteria, fungi, viruses, etc.) for overall taxonomic profiling of the sample. Finally, de novo assembly of the effective reads was performed using metaSPAdes [28] with a minimum contig length of 500 bp to generate contigs. Quality control, Illumina platform sequencing, and sequence assembly were conducted by Chengdu Life Baseline Technology Co., Ltd.

### Genomic characterization of GToV/SWUN/SC

The ORFs of GToV/SWUN/SC were predicted using ORFfinder (https://www.ncbi.nlm.nih.gov/orffinder/) and the three most similar AToV genomes from NCBI. Conserved replicase domains in pp1ab were identified and mutations between GToV and AToV analyzed. Multiple sequence alignment was performed using the ClustalW tool integrated in BioEdit (v7.1). Sequence identity analysis of the ORF1a, ORF1b, S, M, HE, and N genes of the GToV/SWUN/SC strain with those of other toroviruses was conducted using the Similar Analysis module in BioAider [43].

### RT-PCR detection

To investigate the prevalence of goat torovirus (GToV) in China, a Reverse Transcription PCR (RT-PCR) assay was developed to detect GToV, targeting the N gene with a 283 bp amplicon. The assay exhibited a minimum detection limit of 2.02 × 10³ copies/µL and was validated for high specificity and reproducibility using several common diarrheagenic pathogens in goats, including Caprine Kobuvirus, Caprine Enterovirus, Caprine Astrovirus, and Bopivirus. PCR was performed using 2× Rapid Taq Master Mix (Vazyme, China). Primers used were Toro-F (5’ TGCCTTTTCAACCACCAAC 3’) and Toro-R (5’ GCTGTCTCATTTGCCATCAT 3’). The 20 µL reaction included 10 µL Taq polymerase, 6 µL ddH₂O, 1 µL of each primer, and 2 µL template. PCR conditions: 95 °C for 3 min, 35 cycles of 95 °C for 15 s, 54.5 °C for 15 s, 72 °C for 3 s, and a final extension at 72 °C for 5 min. A total of 669 goat fecal samples were tested using the reverse-transcribed cDNA as the template.

### Phylogenetic and recombination analysis

IQ-TREE (https://iqtree.github.io/), a fast and effective stochastic algorithm to estimating maximum-likelihood phylogeny [26], was used. In this study, phylogenetic analysis was conducted using the maximum likelihood method by invoking IQ-TREE (v1.6.12) through the command line. The software employed ModelFinder [18] to automatically select the best-fit substitution model and performed 1000 bootstrap replications. Given that toroviruses are prone to recombination [4, 36], recombination analysis was performed using seven methods: RDP, GeneConv, Chimaera, MaxChi, BootScan, SiScan, and 3Seq in RDP4.0 and SimPlot3.5.1. Recombination events predicted in this study need to be supported by six different methods in RDP4.0 (recombination score > 0.4) and SimPlot 3.5.1 software.

### The analysis of S and HE genes

To investigate potential mutations, the amino acid sequences of the S1 subunit were aligned using the ClustalW algorithm implemented in BioEdit (version 7.1). Using BToV HE protein (SMTL ID: 3i26.1) as a template, 3D models of the HE protein for GToV/SWUN/SC, GToV/SC, and AToV/Qinghai1 were constructed with SWISS-MODEL (https://swissmodel.expasy.org/interactive) and compared using PyMOL. HE gene amino acid sequences were also aligned to identify mutations.

## Results

We obtained a complete torovirus coding sequence using high-throughput sequencing technology, temporarily named GToV/SWUN/SC (GenBank accession number: PQ368548.1). The genomic sequence is 28,457 nucleotides long, with a G + C content of 37.17%. BLAST analysis in the NCBI database revealed that the GToV/SWUN/SC strain shares the highest nucleotide identity (96.73–96.79%) with the three AToV genomes (MZ438674.1, MZ438675.1, MZ438676.1). Consistent with the structure of toroviruses, the entire genome of the GToV/SWUN/SC strain includes six ORFs: ORF1a, ORF1b, S, M, HE, and N (Supplementary Fig. 1 A). ORF1a and ORF1b partially overlap and collectively encode a large replicase polyprotein (pp1ab), which contains 11 conserved domains [8, 33, 38, 42]. Mutation analysis revealed multiple point mutations in these conserved domains (Supplementary Fig. 1B). The comparison of the GToV/SWUN-/SC strain with BToV, PToV, EToV, the unclassified GToV/SZ strain, AToV, and Torovirus sp. is shown Supplementary Table 1. The nucleotide identities between GToV/SWUN/SC and AToV for the S, M, HE, and N genes were 95.83–95.85%, 98.71–98.86%, 98.56%, and 98.97%, respectively; and the amino acid identities were 97.85–97.98%, 100%, 99.04%, and 100%, respectively. In contrast, the nucleotide identities between GToV/SWUN/SC and GToV/SZ for these genes were 73.83%, 79.69%, 70.56%, and 67.47%, and the corresponding amino acid identities were 78.88%, 90.13%, 71.67%, and 66.47%, respectively.

**Fig. 2:**
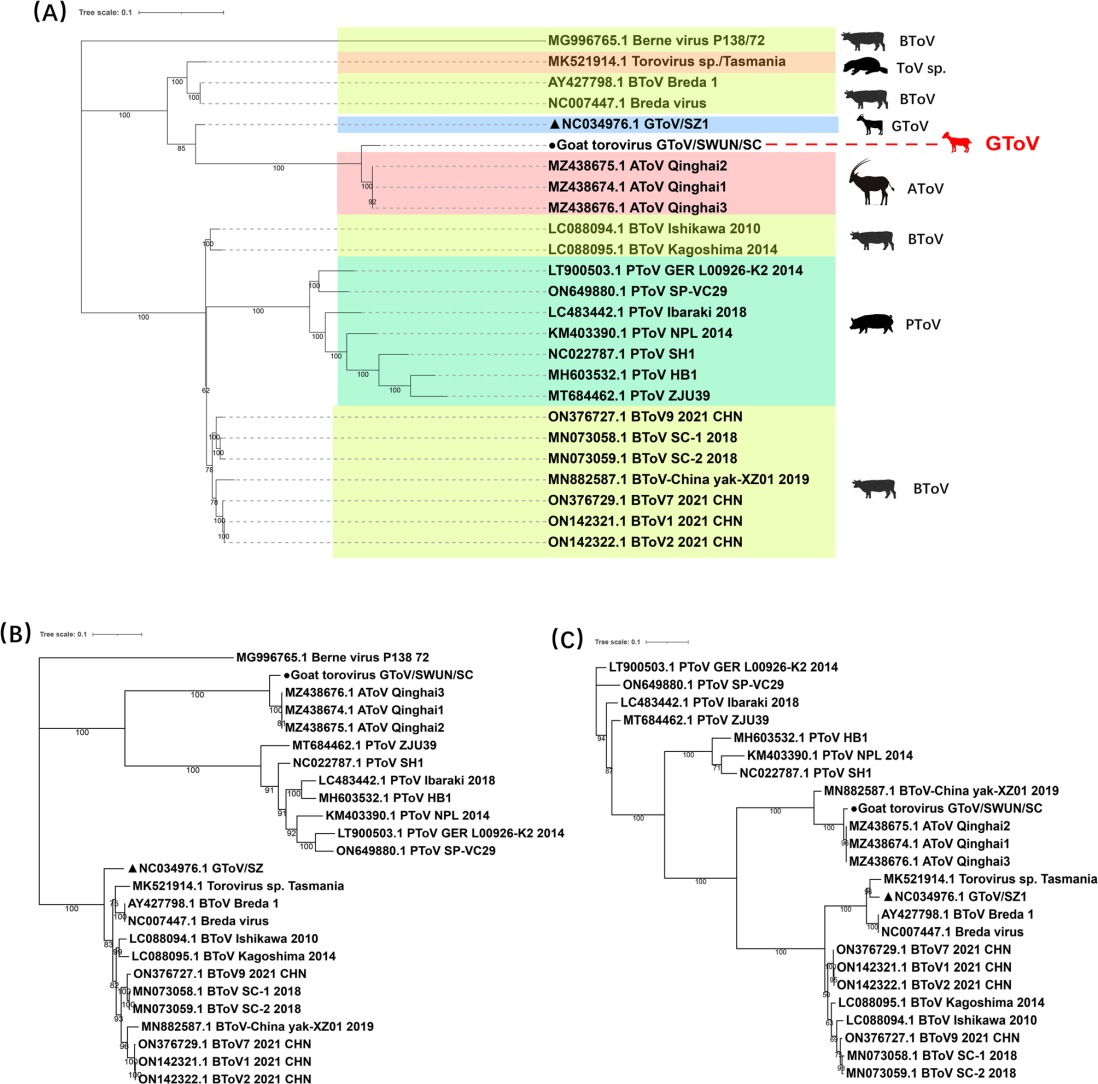
Phylogenetic analysis was conducted using the maximum likelihood method with IQ-TREE software, and 1000 bootstrap replications. **A** For the phylogenetic tree based on the whole genome, the best-fitting model selected by IQ-TREE was GTR + F + I + G4; **B** The phylogenetic tree based on the complete S gene amino acid sequence was constructed using the FLU + F + R3 model; **C** The phylogenetic tree based on the complete HE gene amino acid sequence was constructed using the TN + F + G4 model. “●” represents the strain from this study, and “▲” represents the strain uploaded to NCBI

**Fig. 3:**
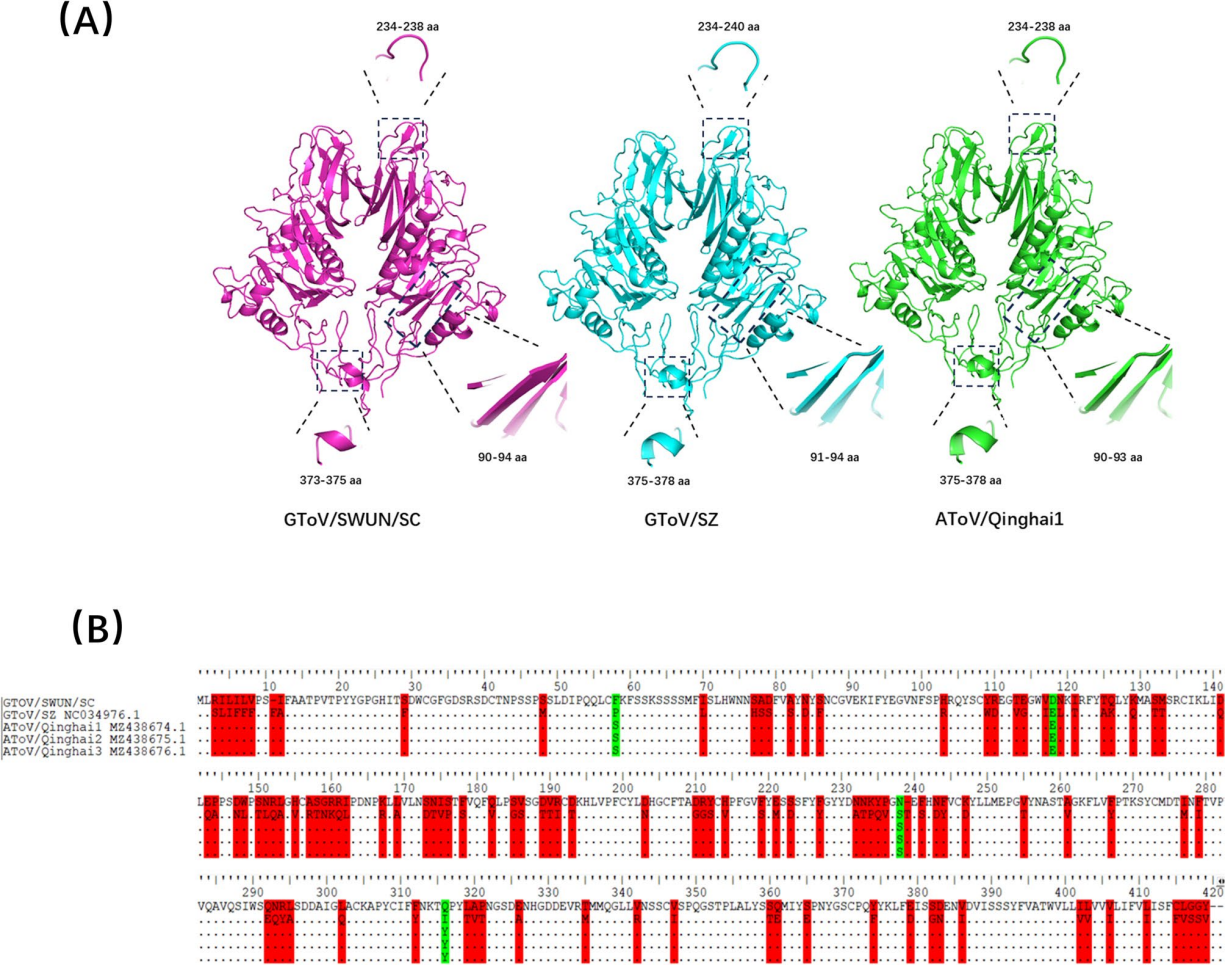
3D Structure Diagram and Mutation Analysis of the HE Gene. **A** Comparison of the 3D structure of the HE gene between AToV and the GToV/SWUN/SC and GToV/SZ strains; **B** Mutation analysis of the HE gene. between the GToV/SWUN/SC strain and AToV. Red indicates amino acid changes in the HE gene of the GToV/SWUN/SC strain compared to the GToV/SZ strain, while green indicates amino acid changes in the HE gene of the GToV/SWUN/SC strain compared to AToV

The phylogenetic tree based on the genome showed that the GToV/SWUN/SC strain forms a distinct large clade with AToV, while it forms two separate clades from the GToV/SZ strain (Fig. 2A). Phylogenetic trees based on the S and HE genes consistently indicated that GToV/SWUN/SC clusters with AToV in a distinct clade, whereas the GToV/SZ strain clusters with BToV and Torovirus sp. in the same branch (Fig. 2B and C). Taken together, these results suggest that GToV/SWUN/SC is more closely related to AToV. Recombination analysis using RDP4.0 and SimPlot3.5.1 software did not predict any recombination events. Analysis of the S and HE genes revealed that the S protein of the GToV/SWUN/SC strain underwent multiple point mutations compared to AToV and GToV/SZ strains (Supplementary Fig. 2). Structural comparison of the HE protein 3D crystal models revealed significant structural variations in three specific regions of the GToV/SWUN/SC strain (Fig. 3A), along with multiple amino acid mutations (Fig. 3B).

The results (Fig. 1) of testing 669 diarrheic fecal samples collected from goats in Sichuan, Chongqing, and Yunnan showed an overall GToV positivity rate of 35.9% (240/669). The positivity rates in Sichuan, Chongqing, and Yunnan were 36.4% (201/552), 32.5% (27/83), and 35.3% (12/34), respectively, indicating that GToV is widely prevalent among goats in these regions further research is needed to assess its global distribution.

## Discussion

In this study, we successfully obtained the complete coding sequence of a goat torovirus strain from diarrheic goat fecal samples, followed by genomic analysis. The GToV/SWUN/SC strain shared a high nucleotide identity with AToV (96.73–96.79%), but only 88.44% identity with the goat-derived GToV/SZ strain. Phylogenetic analysis showed that GToV/SWUN/SC formed a distinct clade with AToV, whereas it formed two separate evolutionary branches with GToV/SZ, indicating a closer genetic relationship between GToV/SWUN/SC and AToV. This suggests a possible recombination event with an unknown torovirus species.

Currently, BToV has been detected in 16 countries, including France, South Africa, Costa Rica, Canada, Venezuela, Hungary, Austria, Japan, South Korea, Brazil, China, Turkey, Uruguay, and Croatia [6, 9, 10, 12, 19, 24, 25, 27, 29, 31, 35, 39]. Porcine torovirus (PToV) has been confirmed to be distributed worldwide and exhibits a high infection rate in pig populations (Hu et al., 2019). Moreover, the newly identified atypical Tibetan antelope torovirus (AToV) has been widely detected in Tibetan antelopes in China [4]. The newly identified GToV strain in this study exhibited a high overall positivity rate, with similarly high positivity rates observed across three different regions. Among the nine goat farms surveyed, only one had no GToV-positive samples. Therefore, GToV may have a broader host range and geographic distribution. In addition to known goat enteric viruses, goat astroviruses, goat Hungarian viruses, and goat Bopivirus, GToV may also be a potential causative agent of diarrhea in goats.

The S protein can be cleaved at the “trypsin-like protease” cleavage site (1003–1007 aa) into two subunits, S1 and S2 [14, 17, 37]. The S1 subunit is responsible for binding to the receptor on the surface of the host cell, while the S2 subunit mediates membrane fusion between the virus and the host cell, playing a crucial role in the viral infection process [23]. The hemagglutinin-esterase (HE) protein is the only viral protein with a resolved crystal structure [21] and is a key determinant of viral host range and adaptability, playing a critical role in viral adaptation to host species. This study found multiple amino acid mutations in the S and HE proteins, as well as structural changes in the HE protein, which may contribute to cross-species transmission of the virus.

Due to the lack of suitable cell lines for torovirus isolation, the biological characteristics of toroviruses remain to be explored. In future studies, we will attempt to isolate and culture the virus. Additionally, the sample distribution in this study is relatively limited; broader surveillance at the national and even global levels is needed to better understand the geographic distribution of the virus. Moreover, the conventional RT-PCR method used in this study has relatively low sensitivity; therefore, we plan to establish a more sensitive real-time quantitative PCR assay for future monitoring.

## Supplementary Information

Supplementary Material 1.

Supplementary Material 2.

Supplementary Material 3.

## Data Availability

No datasets were generated or analysed during the current study.

## References

[1] Aita T, Kuwabara M, Murayama K, Sasagawa Y, Yabe S, Higuchi R, Tamura T, Miyazaki A, Tsunemitsu H. Characterization of epidemic diarrhea outbreaks associated with bovine torovirus in adult cows. Arch Virol. 2012;157:423–31. 10.1007/s00705-011-1183-9. 22167249 10.1007/s00705-011-1183-9 PMC7087103

[2] Brown DW, Beards GM, Flewett TH. Detection of Breda virus antigen and antibody in humans and animals by enzyme immunoassay. J Clin Microbiol. 1987;25:637–40. 10.1128/jcm.25.4.637-640.1987. 3571473 10.1128/jcm.25.4.637-640.1987 PMC266050

[3] Brown DW, Selvakumar R, Daniel DJ, Mathan VI. Prevalence of neutralising antibodies to Berne virus in animals and humans in vellore, South india. Brief report. Arch Virol. 1988;98:267–9. 10.1007/BF01322174. 3348749 10.1007/BF01322174 PMC7086926

[4] Dai X, Lu S, Shang G, Zhu W, Yang J, Liu L, Xu J. Characterization and identification of a novel torovirus associated with recombinant bovine torovirus from Tibetan antelope in Qinghai-Tibet plateau of China. Front Microbiol. 2021;12:737753. 10.3389/fmicb.2021.737753. 34552576 10.3389/fmicb.2021.737753 PMC8451951

[5] Draker R, Roper RL, Petric M, Tellier R. The complete sequence of the bovine torovirus genome. Virus Res. 2006;115:56–68. 10.1016/j.virusres.2005.07.005. 16137782 10.1016/j.virusres.2005.07.005 PMC7114287

[6] Duckmanton L, Carman S, Nagy E, Petric M. Detection of bovine torovirus in fecal specimens of calves with diarrhea from Ontario farms. J Clin Microbiol. 1998;36:1266–70. 10.1128/JCM.36.5.1266-1270.1998. 9574689 10.1128/jcm.36.5.1266-1270.1998 PMC104812

[7] Gallagher TM, Buchmeier MJ. Coronavirus spike proteins in viral entry and pathogenesis. Virology. 2001;279:371–4. 10.1006/viro.2000.0757. 11162792 10.1006/viro.2000.0757 PMC7133764

[8] Gorbalenya AE, Enjuanes L, Ziebuhr J, Snijder EJ. Nidovirales: evolving the largest RNA virus genome. Virus Res. 2006;117:17–37. 10.1016/j.virusres.2006.01.017. 16503362 10.1016/j.virusres.2006.01.017 PMC7114179

[9] Gülaçtı I, Işıdan H, Sözdutmaz I. Detection of bovine torovirus in fecal specimens from calves with diarrhea in Turkey. Arch Virol. 2014;159:1623–7. 10.1007/s00705-014-1977-7. 24420162 10.1007/s00705-014-1977-7

[10] Haschek B, Klein D, Benetka V, Herrera C, Sommerfeld-Stur I, Vilcek S, Moestl K, Baumgartner W. Detection of bovine torovirus in neonatal calf diarrhoea in lower Austria and Styria (Austria). J Vet Med B Infect Dis Vet Public Health. 2006;53:160–5. 10.1111/j.1439-0450.2006.00936.x. 16629982 10.1111/j.1439-0450.2006.00936.x PMC7165904

[11] Hoet AE, Chang K-O, Saif LJ. Comparison of ELISA and RT-PCR versus immune electron microscopy for detection of bovine torovirus (Breda virus) in calf fecal specimens. J Vet Diagn Invest. 2003;15:100–6. 10.1177/104063870301500203. 12661719 10.1177/104063870301500203

[12] Hoet AE, Cho K-O, Chang K-O, Loerch SC, Wittum TE, Saif LJ. Enteric and nasal shedding of bovine torovirus (Breda virus) in feedlot cattle. Am J Vet Res. 2002;63(3):342–8. 10.2460/ajvr.2002.63.342. 11911568 10.2460/ajvr.2002.63.342

[13] Hoet AE, Saif LJ. Bovine torovirus (Breda virus) revisited. Anim Health Res Rev. 2004;5:157–71. 10.1079/ahr200498. 15984322 10.1079/ahr200498

[14] Horzinek MC, Ederveen J, Kaeffer B, de Boer D, Weiss M. The peplomers of Berne virus. J Gen Virol. 1986;67:2475–83. 10.1099/0022-1317-67-11-2475. 3783129 10.1099/0022-1317-67-11-2475

[15] Hosmillo MDT, Jeong Y-J, Kim H-J, Collantes TM, Alfajaro MM, Park J-G, Kim H-H, Kwon H-J, Park S-J, Kang M-I, Park S-I, Cho K-O. Development of universal SYBR green real-time RT-PCR for the rapid detection and quantitation of bovine and Porcine toroviruses. J Virol Methods. 2010;168:212–7. 10.1016/j.jviromet.2010.06.001. 20558206 10.1016/j.jviromet.2010.06.001 PMC7112831

[16] Hu Z-M, Yang Y-L, Xu L-D, Wang B, Qin P, Huang Y-W. Porcine torovirus (PToV)-A brief review of etiology, diagnostic assays and current epidemiology. Front Vet Sci. 2019a;6: 120. 10.3389/fvets.2019.00120. 31058174 10.3389/fvets.2019.00120 PMC6482245

[17] Hu Z-M, Yang Y-L, Xu L-D, Wang B, Qin P, Huang Y-W. Porcine torovirus (PToV)—A brief review of etiology, diagnostic assays and current epidemiology. Front Vet Sci. 2019;6. 10.3389/fvets.2019.00120. 10.3389/fvets.2019.00120 PMC6482245 31058174

[18] Kalyaanamoorthy S, Minh BQ, Wong TKF, von Haeseler A, Jermiin LS. Modelfinder: fast model selection for accurate phylogenetic estimates. Nat Methods. 2017;14:587–9. 10.1038/nmeth.4285. 28481363 10.1038/nmeth.4285 PMC5453245

[19] Kirisawa R, Takeyama A, Koiwa M, Iwai H. Detection of bovine torovirus in fecal specimens of calves with diarrhea in Japan. J Vet Med Sci. 2007;69:471–6. 10.1292/jvms.69.471. 17551218 10.1292/jvms.69.471

[20] Koopmans M, van den Boom U, Woode G, Horzinek MC. Seroepidemiology of Breda virus in cattle using ELISA. Vet Microbiol. 1989;19:233–43. 10.1016/0378-1135(89)90069-2. 2718353 10.1016/0378-1135(89)90069-2 PMC7117312

[21] Langereis MA, Zeng Q, Gerwig GJ, Frey B, von Itzstein M, Kamerling JP, de Groot RJ, Huizinga EG. Structural basis for ligand and substrate recognition by torovirus hemagglutinin esterases. Proc Natl Acad Sci U S A. 2009;106:15897–902. 10.1073/pnas.0904266106. 19721004 10.1073/pnas.0904266106 PMC2747215

[22] Langmead B, Salzberg SL. Fast gapped-read alignment with bowtie 2. Nat Methods. 2012;9:357–9. 10.1038/nmeth.1923. 22388286 10.1038/nmeth.1923 PMC3322381

[23] Li F. Structure, function, and evolution of coronavirus Spike proteins. Annu Rev Virol. 2016;3:237–61. 10.1146/annurev-virology-110615-042301. 27578435 10.1146/annurev-virology-110615-042301 PMC5457962

[24] Liebler EM, Klüver S, Pohlenz J, Koopmans M. [The significance of Bredavirus as a diarrhea agent in calf herds in lower saxony]. Dtsch Tierarztl Wochenschr. 1992;99:195–200. 1322268

[25] Lojkić I, Krešić N, Šimić I, Bedeković T. Detection and molecular characterisation of bovine corona and toroviruses from Croatian cattle. BMC Vet Res. 2015;11:202. 10.1186/s12917-015-0511-9. 26268320 10.1186/s12917-015-0511-9 PMC4535285

[26] Nguyen L-T, Schmidt HA, von Haeseler A, Minh BQ. IQ-tree: a fast and effective stochastic algorithm for estimating maximum-likelihood phylogenies. Mol Biol Evol. 2015;32:268–74. 10.1093/molbev/msu300. 25371430 10.1093/molbev/msu300 PMC4271533

[27] Nogueira JS, Asano KM, de Souza SP, Brandão PE, Richtzenhain LJ. First detection and molecular diversity of Brazilian bovine torovirus (BToV) strains from young and adult cattle. Res Vet Sci. 2013;95:799–801. 10.1016/j.rvsc.2013.04.006. 23648077 10.1016/j.rvsc.2013.04.006 PMC7111811

[28] Nurk S, Meleshko D, Korobeynikov A, Pevzner PA. Metaspades: a new versatile metagenomic assembler. Genome Res. 2017;27:824–34. 10.1101/gr.213959.116. 28298430 10.1101/gr.213959.116 PMC5411777

[29] Park S-J, Oh E-H, Park S-I, Kim H-H, Jeong Y-J, Lim G-K, Hyun B-H, Cho K-O. Molecular epidemiology of bovine toroviruses circulating in South Korea. Vet Microbiol. 2008;126:364–71. 10.1016/j.vetmic.2007.07.012. 17719729 10.1016/j.vetmic.2007.07.012 PMC7117412

[30] Peng G, Xu L, Lin Y-L, Chen L, Pasquarella JR, Holmes KV, Li F. Crystal structure of bovine coronavirus spike protein lectin domain. J Biol Chem. 2012;287:41931–8. 10.1074/jbc.M112.418210. 23091051 10.1074/jbc.M112.418210 PMC3516740

[31] Pérez E, Kummeling A, Janssen MM, Jiménez C, Alvarado R, Caballero M, Donado P, Dwinger RH. Infectious agents associated with diarrhoea of calves in the Canton of Tilarán, Costa Rica. Prev Vet Med. 1998;33:195–205. 10.1016/s0167-5877(97)00038-x. 9500174 10.1016/S0167-5877(97)00038-X PMC7134171

[32] Pradesh U, Vishwa PDDUPC, CrnrK V, Izcitncigcir D, B. Toroviruses affecting animals and humans: A review. Asian J Anim Veterinary Adv. 2014;9:190–201.

[33] Saberi A, Gulyaeva AA, Brubacher JL, Newmark PA, Gorbalenya AE. A planarian nidovirus expands the limits of RNA genome size. PLoS Pathog. 2018;14:e1007314. 10.1371/journal.ppat.1007314. 30383829 10.1371/journal.ppat.1007314 PMC6211748

[34] Shin D-J, Park S-I, Jeong Y-J, Hosmillo M, Kim H-H, Kim H-J, Kwon H-J, Kang M-I, Park S-J, Cho K-O. Detection and molecular characterization of Porcine toroviruses in Korea. Arch Virol. 2010;155:417–22. 10.1007/s00705-010-0595-2. 20127374 10.1007/s00705-010-0595-2 PMC7087203

[35] Smits SL, Lavazza A, Matiz K, Horzinek MC, Koopmans MP, De Groot RJ. Phylogenetic and evolutionary relationships among torovirus field variants: evidence for multiple intertypic recombination events. J Virol. 2003;77:9567–77. 10.1128/JVI.77.17.9567-9577.2003. 12915570 10.1128/JVI.77.17.9567-9577.2003 PMC187415

[36] Ujike M, Taguchi F. Recent progress in torovirus molecular biology. Viruses. 2021a;13:435. 10.3390/v13030435. 33800523 10.3390/v13030435 PMC7998386

[37] Ujike M, Taguchi F. Recent progress in torovirus molecular biology. Viruses. 2021b;13:435. 10.3390/v13030435. 33800523 10.3390/v13030435 PMC7998386

[38] van Boheemen S, de Graaf M, Lauber C, Bestebroer TM, Raj VS, Zaki AM, Osterhaus ADME, Haagmans BL, Gorbalenya AE, Snijder EJ, Fouchier RAM. Genomic characterization of a newly discovered coronavirus associated with acute respiratory distress syndrome in humans. mBio. 2012;3:e00473–12. 10.1128/mBio.00473-12. 23170002 10.1128/mBio.00473-12 PMC3509437

[39] Vorster JH, Gerdes GH. Breda virus-like particles in calves in South Africa. J S Afr Vet Assoc. 1993;64:58. 8410941

[40] Weiss M, Steck F, Kaderli R, Horzinek MC. Antibodies to Berne virus in horses and other animals. Vet Microbiol. 1984;9:523–31. 10.1016/0378-1135(84)90014-2. 6506447 10.1016/0378-1135(84)90014-2 PMC7117441

[41] Wood DE, Lu J, Langmead B. Improved metagenomic analysis with kraken 2. Genome Biol. 2019;20:257. 10.1186/s13059-019-1891-0. 31779668 10.1186/s13059-019-1891-0 PMC6883579

[42] Zeng C, Wu A, Wang Y, Xu S, Tang Y, Jin X, Wang S, Qin L, Sun Y, Fan C, Snijder EJ, Neuman BW, Chen Y, Ahola T, Guo D. Identification and characterization of a ribose 2’-O-methyltransferase encoded by the ronivirus branch of nidovirales. J Virol. 2016;90:6675–85. 10.1128/JVI.00658-16. 27170751 10.1128/JVI.00658-16 PMC4944298

[43] Zhou Z-J, Qiu Y, Pu Y, Huang X, Ge X-Y. Bioaider: an efficient tool for viral genome analysis and its application in tracing SARS-CoV-2 transmission. Sustain Cities Soc. 2020;63: 102466. 10.1016/j.scs.2020.102466. 32904401 10.1016/j.scs.2020.102466 PMC7455202

