# Supplementary material for: Identification of a new goat torovirus strain: first detection and genomic analysis in China
Source: Ir Vet J. 2025 Aug 27;78:20. doi: 10.1186/s13620-025-00305-3 (PMC12382054; doi:10.1186/s13620-025-00305-3)
Supplement: Supplementary file 1 — Supplementary Material 1. [file 13620_2025_305_MOESM1_ESM.docx]

**Supplementary Table 1** **The nucleotide and amino acid identity between the GToV/SWUN/SC strain and other toroviruses**

|  | **Nucleotide identity (%)** | | | | | | |
| --- | --- | --- | --- | --- | --- | --- | --- |
|  | **Genome** | **ORF1a** | **ORF1b** | **S** | **M** | **HE** | **N** |
| **BToV** | 82.30-85.26 | 76.5-89.1 | 27.42-86.17 | 73.52-74.11 | 23.47-78.97 | 71.12-91.55 | 65.87-77.3 |
| **PToV** | 79.12-81.93 | 76.23-76.82 | 30.24-86.51 | 73.66-74.23 | 79.69-82.40 | 68.45-70.09 | 75.46-77.71 |
| **EToV** | 80.0 | 65.22 | 51.31 | 71.14 | 78.11 | 21.98 | 22.46 |
| **AToV** | 96.72-96.78 | 96.5-96.6 | 97.06-97.12 | 95.83-95.85 | 98.71-98.86 | 98.56 | 98.97 |
| **GToV/SZ** | 88.43 | 94.07 | 80.74 | 73.83 | 79.69/ | 70.56 | 67.47 |
| **Torovirus sp.** | 84.84 | 88.34 | 78.63 | 73.92 | 78.83 | 70.49 | 66.67 |
|  | **Amino acid identity (%)** | | | | | | |
|  |  | **ORF1a** | **ORF1b** | **S** | **M** | **HE** | **N** |
| **BToV** |  | 77.16-91.27 | 30.01-92.67 | 78.06-78.94 | 10.98-91.42 | 70.41-91.63 | 65.27-79.75 |
| **PToV** |  | 76.54-77.03 | 7.41-92.71 | 76.75-77.89 | 89.70-90.99 | 65.35-68.06 | 75.46-77.91 |
| **EToV** |  | 62.34 | 55.39 | 74.15 | 87.98 | 9.59 | 9.01 |
| **AToV** |  | 97.1-97.25 | 98.52-98.65 | 97.85-97.98 | 100 | 99.04 | 100 |
| **GToV/SZ** |  | 95.39 | 88.61 | 78.88 | 90.13 | 71.67 | 66.47 |
| **Torovirus sp.** |  | 89.97 | 85.95 | 78.94 | 90.99 | 70.17 | 65.27 |
