# Supplementary figures and images for: Identification of a new goat torovirus strain: first detection and genomic analysis in China
Source: Ir Vet J. 2025 Aug 27;78:20. doi: 10.1186/s13620-025-00305-3 (PMC12382054; doi:10.1186/s13620-025-00305-3)

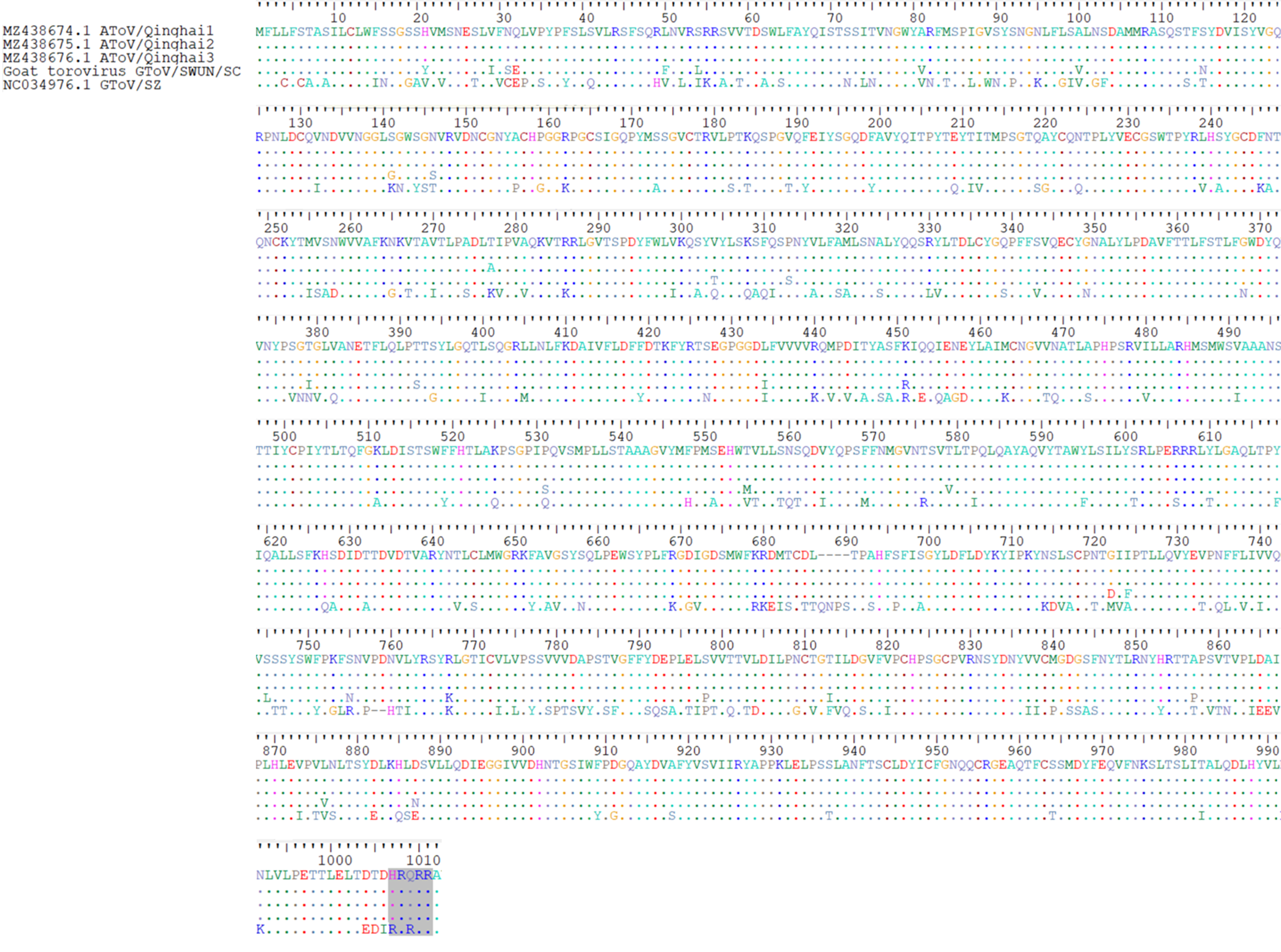

Supplement: Supplementary file 3 — Supplementary Material 3. [file 13620_2025_305_MOESM3_ESM.tif]
